# Proof-of-concept for an automatable mortality prediction scoring in hospitalised older adults

**DOI:** 10.3389/fmed.2024.1329107

**Published:** 2024-05-23

**Authors:** Vanda W. T. Ho, Natalie M. W. Ling, Denishkrshna Anbarasan, Yiong Huak Chan, Reshma Aziz Merchant

**Affiliations:** ^1^Division of Geriatric Medicine, Department of Medicine, National University Health System, Singapore, Singapore; ^2^Department of Medicine, Yong Loo Lin School of Medicine, National University of Singapore, Singapore, Singapore; ^3^Biostatistics Unit, Yong Loo Lin School of Medicine, National University of Singapore, Singapore, Singapore

**Keywords:** older adults, hospitalisation, survivorship, mortality, predictive tool

## Abstract

**Introduction:**

It is challenging to prognosticate hospitalised older adults. Delayed recognition of end-of-life leads to failure in delivering appropriate palliative care and increases healthcare utilisation. Most mortality prediction tools specific for older adults require additional manual input, resulting in poor uptake. By leveraging on electronic health records, we aim to create an automatable mortality prediction tool for hospitalised older adults.

**Methods:**

We retrospectively reviewed electronic records of general medicine patients ≥75 years at a tertiary hospital between April–September 2021. Demographics, comorbidities, ICD-codes, age-adjusted Charlson Comorbidity Index (CCI), Hospital Frailty Risk Score, mortality and resource utilization were collected. We defined early deaths, late deaths and survivors as patients who died within 30 days, 1 year, and lived beyond 1 year of admission, respectively. Multivariate logistic regression analyses were adjusted for age, gender, race, frailty, and CCI. The final prediction model was created using a stepwise logistic regression.

**Results:**

Of 1,224 patients, 168 (13.7%) died early and 370 (30.2%) died late. From adjusted multivariate regression, risk of early death was significantly associated with ≥85 years, intermediate or high frail risk, CCI > 6, cardiovascular risk factors, AMI and pneumonia. For late death, risk factors included ≥85 years, intermediate frail risk, CCI >6, delirium, diabetes, AMI and pneumonia. Our mortality prediction tool which scores 1 point each for age, pneumonia and AMI had an AUC of 0.752 for early death and 0.691 for late death.

**Conclusion:**

Our mortality prediction model is a proof-of-concept demonstrating the potential for automated medical alerts to guide physicians towards personalised care for hospitalised older adults.

## Introduction

Older adults account for an increasing proportion of patients on the general or geriatric medicine floor (1). These patients have a higher degree of clinical complexity, multimorbidity defined by the presence of two or more chronic health conditions (2) and geriatric syndromes such as dementia and frailty which is a state of “reduced physiologic reserve that increases an individual’s vulnerability to adverse events when exposed to stressors” (3). Unlike their younger counterparts, a significant portion of frail older patients will experience a decline in function during their hospital admission and about a-third would die within the year from discharge (4). Clearly, care for older adults needs to be personalised, tailored to their preferences, functional trajectory, and life expectancy. However, due to practical and logistical constraints, most hospitals do not have sufficient geriatricians for all older patients and to fill the void, care is often delivered by other specialists (5). Frail or at risk older patients tend to have unpredictable end of life trajectory, and prognosticating death can be difficult for physicians who are not familiar with care of older adults (6). In acute hospitals where the culture leans towards curative intent, this default approach may not be appropriate or indeed achievable in many cases. As a result, many older patients have increased use of healthcare resources towards their end of life, yet, they do not experience a good death. A good death in older adult is recognised as a priority worldwide in recent years (7). Many countries recognise that majority of frail older people are dying in acute hospitals (8, 9) and have started rolling out nation-wide schemes to improve the death experience (10).

Many healthcare systems have rolled out “Choosing Wisely” initiatives (11) as less is often more in these group of patients. Most studies on time to benefit from specified therapeutics excluded these very group of patients. Appropriate therapeutic approaches depend on the balance between life expectancy and time to benefit from the therapy. Mortality prediction tools are useful in directing the physician towards providing quality personalised care that integrates patient preference, active management, and palliative care (12). Several mortality prediction tools for older patients have been developed but they require manual input from physicians (13, 14), which precludes them from widespread implementation. The bottleneck remains the physicians’ awareness to consider prognosis before determining the care approach. Additionally, many standard prediction tools in clinical practice are developed for the general population and do not cater specifically to older patients with variable trajectory, multimorbidity, functional and psychosocial issues (15, 16).

Frailty has been associated with mortality in older adults (17, 18), with a relative risk of 1.6 to 3.1 for 90-day mortality in acute medical units (19), and may be a potential predictor for mortality. However, studies have shown that frailty alone is insufficiently accurate (19). The Hospital Frailty Risk Score (HFRS), which uses data from electronic medical records such as demographic, comorbidity and prior hospitalisation information to derive risk of frailty, has been previously validated for 30-day mortality outcomes in the acute care setting (20) and older patients with heart failure (21), but not in critical care (22). It demonstrates that with the advent of electronic medical records, we can create prediction scores leveraging on automated medical alerts to prompt physicians towards appropriate care for older adults. As many older adults may not die that acutely, these tools may guide physicians in planning proper care transitions and follow up, and could serve as a communication tool between different providers. Hence, a mortality prediction tool that can predict a longer timepoint is sorely needed. Therefore, from a retrospective review of our hospital’s electronic medical records, we aim to investigate the differing risk factors associated with mortality and create an automatable prediction tool for mortality in hospitalised older adults within the year following discharge.

## Materials and methods

### Database and study population

We conducted a retrospective cohort study on 1,224 older adults aged 75 years and older who were admitted to the Internal Medicine service at the National University Hospital, a 1,239-bed tertiary hospital located in the western region of Singapore, between April and September 2021. These patients were managed by both geriatricians and non-geriatricians. Patients admitted to the Acute Medical Unit and isolation wards were excluded from the study.

### Data collection and outcomes

We collected patient’s demographic information, comorbidities, primary diagnoses, age-adjusted Charlson Comorbidity Index (CCI) and HFRS. The age-adjusted CCI is a validated scoring system comprising a weighted index of age, number, and seriousness of comorbid disease which has shown to be predictive of mortality. HFRS, which generates a frailty risk score from ICD-10 codes, has been validated to be predictive of length of stay, inpatient mortality, adverse events, and costs (5). A score of <5 indicates low frailty risk, 5–15 indicates intermediate risk, and > 15 indicates high frailty risk. From our data on time to mortality, we defined survivorship to be: early death for mortality within 30 days of admission, late deaths for mortality between 30 days and 1 year of admission, and survivors who were alive 1 year after admission. Albumin levels, which have been found in previous studies to be predictive of mortality risk (23–25), were recorded. We also collected indices of resource utilization such as opioid use, number of hospital admissions in the year preceding death, and cost during their final admission.

### Statistical analysis

Stata Version 17.0 was used for analysis with statistical significance set at *p* < 0.05. Descriptive analyses were presented as frequencies with percentages for categorical variables, and mean with SD or median with interquartile range for continuous variables. Significance testing by Pearson χ2 test for categorical and Mann–Whitney U tests for continuous variables were conducted. Risk predictors for survivorship as the primary outcome (early mortality within 30 days of admission and late mortality beyond 30 days to 1 year of admission) were investigated using Multivariate logistic regression. Odds ratios (OR) with 95% confidence intervals (95% CI) were presented.

Variables that were significant from the multivariate logistic regression for both early and late mortality were included in the risk score model. Logistic regression for a binary outcome was purposefully chosen for creation of a pragmatic tool which reflects the binary nature of decisions in clinical practice. Often, clinicians would decide if patient is close to end of life based on the surprise question (26), is patient likely to die in the next 6 months. Conversely, using Cox regression and thinking in terms of time to mortality is harder to do clinically and difficult to prognosticate accurately. Utility of the model was assessed with sensitivity, specificity, positive and negative predictive values. These parameters were selected to aid the confidence of the clinician in forming sensible predictions for each cut-off. Receiver Operating Curve (ROC) analysis was constructed to evaluate the discriminative ability of the prediction model using a 1-point score for each positive predictor.

## Results

A total of 1,224 patients were included in the study. Baseline characteristics according to time to mortality from admission are summarized in Table 1. The mean age was 84.7 ± 6.2 years, with 712 (58.2%) being female and 984 (80.4%) of Chinese ethnicity. Amongst the 370 patients who died within one year, 202 patients (54.6%) had late deaths, and 168 (45.4%) had early deaths. Amongst the early deaths, 105 (62.5%) occurred during inpatient stay, and remaining 65 (37.5%) within 30 days of discharge (Supplementary Table S1).

**Table 1:** Demographics by time to mortality from discharge within 1 year.

	All	Time to mortality from discharge		
		Within 30 days (early deaths)	After 30 days (late deaths)	No mortality (survivors)
	*N* = 1,224	*n* = 168 (13.7%)	*n* = 202 (16.5%)	*n* = 854 (69.8%)
Demographics				
Gender				
Male	512 (41.8)	80 (47.6)	902 (45.5)	340 (39.8)
Female	712 (58.2)	88 (52.4)	110 (54.5)	514 (60.2)
Age (years)	84.67 ± 6.19	87.60 ± 6.53^a^	86.29 ± 6.48^b^	83.71 ± 5.79^a,b^
Ethnicity				
Chinese	984 (80.4)	142 (84.5)	163 (80.7)	679 (79.5)
Malay	102 (8.3)	14 (8.3)	17 (8.5)	71 (8.3)
Indian	80 (6.5)	7 (4.2)	13 (6.5)	60 (7.0)
Others	58 (4.7)	5 (3.0)	9 (4.5)	44 (5.2)
Diagnosis				
Pneumonia	358 (29.2)	110 (65.5)	66 (32.7)	182 (21.3)
Delirium	357 (29.2)	66 (39.3)	77 (38.1)	214 (25.1)
Fragility fracture	59 (4.8)	4 (2.4)	11 (5.4)	44 (5.2)
Urinary tract infection	391 (31.9)	50 (29.8)	77 (38.1)	264 (30.9)
Stroke	14 (1.1)	1 (0.6)	3 (1.5)	10 (1.2)
Intracranial bleed	27 (2.2)	2 (1.2)	7 (3.5)	18 (2.1)
Acute myocardial infarction	98 (8.0)	34 (20.2)	23 (11.4)	41 (4.8)
Comorbidities				
Diabetes	512 (42.6)	70 (41.7)	87 (43.1)	364 (42.6)
Hypertension	547 (44.7)	74 (44.0)	92 (45.5)	381 (44.6)
Hyperlipidaemia	437 (35.7)	55 (32.7)	74 (36.6)	308 (36.1)
Dementia	144 (11.8)	26 (15.5)	22 (10.9)	96 (11.2)
Chronic kidney disease	389 (31.8)	69 (41.1)	81 (40.1)	239 (28.0)
Serum albumin	33.73 ± 5.36	29.14 ± 5.31^a,b^	31.72 ± 5.18^b,c^	34.95 ± 4.84^a,c^
Hospital frailty risk score (median (IQR))	5.70 (9.20)	7.80 (12.10)^a^	6.85 (9.60)^b^	4.90 (8.40)^a,b^
Low	580 (47.4)	61 (36.3)	80 (40.0)	439 (51.3)
Intermediate	484 (39.5)	72 (42.9)	92 (46.0)	320 (37.4)
High	160 (13.1)	35 (20.8)	28 (14.0)	97 (11.3)
Age adjusted Charlson’s comorbidity index (median (IQR))				
Tertile 1	532 (43.5)	58 (34.5)	72 (35.6)	402 (47.1)
Tertile 2	284 (23.2)	37 (22.0)	47 (23.3)	200 (23.4)
Tertile 3	408 (33.3)	73 (43.5)	83 (41.1)	252 (29.5)
Fentanyl	59 (4.8)	21 (12.5)	38 (18.8)	0 (0.0)
Morphine	24 (2.0)	9 (5.4)	15 (7.4)	0 (0.0)
Outcomes				
Number of admissions in past 1 year				
Mean	1.11 ± 1.79	1.51 ± 1.76^a^	1.47 ± 2.35^b^	0.94 ± 1.61^a,b^
Median (IQR)	1.00 (2.00)	1.00 (2.00)^a^	1.00 (2.00)^b^	0.00 (1.00)^a,b^
Length of stay in final admission (Days)				
Mean	7.25 ± 7.09	10.20 ± 9.15^a,b^	7.82 ± 7.25^a^	6.54 ± 6.40^b^
Median (IQR)	5.00 (6.00)	7.00 (10.00)^a,b^	6.00 (5.00)^a^	5.00 (5.00)^b^
Total cost ($)				
Mean	5983.78 ± 5021.97	7990.99 ± 6939.42^a,b^	6317.10 ± 4787.24^a^	5512.32 ± 4500.57^b^
Median (IQR)	4522.73 (4600.30)	5752.12 (7779.86)^a^	4966.03 (4370.68)	4124.90 (4172.76)^a^

Values presented as *n* (%) or mean ± SD or median (IQR) whenever specified; Bold indicates significant difference (*p* < 0.05); ^abc^Values with common superscript alphabet are significantly different.

Non-survivors tended to be older (87.6 ± 6.53 and 86.29 ± 6.48 years vs. 83.71 ± 5.79 years, *p* < 0.001), have a diagnosis of pneumonia (65.5 and 32.7% vs. 21.3%, *p* < 0.001), delirium (39.3 and 38.1% vs. 25.1%, *p* < 0.001) and myocardial infarction (20.2 and 11.4% vs. 4.8%, *p* < 0.001) compared to those who survived. Median HFRS scores were significantly higher for those who died, compared to those who survived (7.80, IQR 12.10 and 6.85, IQR 9.60 vs. 5.70, IQR 9.20). In terms of resource utilisation at end-of-life, opiate use was higher among the late death group as compared to the early death group, with 18.8% using fentanyl (vs 12.5%, *p* < 0.001) and 7.4% using morphine (vs 5.4%, *p* < 0.001). Patients who had early death had more admissions in the year leading up to death (1.51 ± 1.76 days vs. 1.47 ± 2.35 days, *p* < 0.001), a longer mean length of stay (LOS) in their final admission (10.20 ± 9.15 days vs. 7.82 ± 7.25 days, *p* < 0.001) and higher mean cost per admission (7990.99 ± 6939.42 SGD vs. 6317.10 ± 4784.24 SGD, *p* < 0.001) compared to those with late deaths. Within the early death group, patients who died within 30 days had longer mean LOS compared to those who died within inpatient (13.00 ± 9.92 days vs. 8.51 ± 8.25 days, *p* = 0.003), and higher mean cost per admission (9838.17 ± 7624.65 vs. 6882.69 ± 6273.92 SGD, *p* = 0.007). Further details are in Supplementary Table S1.

For early deaths compared to survivors, univariate logistic regression found age ≥ 85 years, intermediate and high frail risk, CCI score of more than 6, delirium, chronic kidney disease (CKD), acute myocardial infarction (AMI) and pneumonia to be significantly associated with increased risk of mortality. In the adjusted multivariate logistic regression analysis, factors that were significantly associated with an increased risk of early death included age ≥ 85 years (OR 3.04, 9,595% CI 2.13–4.36), being at intermediate (OR 1.55, 95% CI 1.06–2.28) and high (OR 1.76, 95% CI 1.07–2.91) frail risk, CCI score of more than 6 (OR 2.09, 95% CI 1.39–3.13), AMI (OR 3.50, 95% CI 2.06–5.96) and pneumonia (OR 6.57, 95% CI 4.52–9.56) (Table 2).

**Table 2:** Associations of time to mortality from discharge within 1 year.

Predictors	Mortality within 30 days post discharge		Mortality from 31 days to 365 days post discharge	
	Unadjusted OR (95% CI); *p* value	Adjusted OR (95% CI); *p* value	Unadjusted OR (95% CI); *p* value	Adjusted OR (95% CI); *p* value
Age ≥ 85 years	**2.96 (2.09–4.20); *p* < 0.001**	**3.04 (2.13–4.36); *p* < 0.001**	**1.96 (1.44–2.68); *p* < 0.001**	**2.04 (1.48–2.80); *p* < 0.001**
Intermediate frail risk^+^	**1.62 (1.12–2.35); *p* = 0.011**	**1.55 (1.06–2.28); *p* = 0.025**	**1.58 (1.13–2.20); *p* = 0.007**	**1.52 (1.08–2.14); *p* = 0.015**
High frail risk^+^	**2.60 (1.62–4.16); *p* < 0.001**	**1.76 (1.07–2.91); *p* = 0.027**	1.58 (0.98–2.57); *p* = 0.062	1.18 (0.71–1.95); *p* = 0.519
Charlsons comorbidity index tertile 2^^^	1.28 (0.82–2.00); *p* = 0.279	1.39 (0.87–2.20); *p* = 0.166	1.30 (0.86–1.95); *p* = 0.209	1.39 (0.92–2.11); *p* = 0.121
Charlsons comorbidity index tertile 3^^^	**2.01 (1.38–2.94); *p* < 0.001**	**2.09 (1.39–3.13); *p* < 0.001**	**1.87 (1.31–2.66); *p* < 0.001**	**1.98 (1.37–2.87); *p* < 0.001**
Dementia	1.45 (0.91–2.32); *p* = 0.121	0.63 (0.35–1.13); *p* = 0.118	0.98 (0.60–1.60); *p* = 0.931	0.57 (0.32–1.02); *p* = 0.059
Delirium	**1.93 (1.37–2.73); *p* < 0.001**	1.31 (0.88–1.95); *p* = 0.183	**1.83 (1.32–2.53); *p* < 0.001**	**1.55 (1.08–2.23); *p* = 0.017**
Intracranial haemorrhage	0.56 (0.13–2.44); *p* = 0.441	0.44 (0.10–1.98); *p* = 0.286	1.69 (0.70–4.10); *p* = 0.247	1.51 (0.61–3.75); *p* = 0.379
Stroke	0.51 (0.06–3.98); *p* = 0.518	0.24 (0.03–1.99); *p* = 0.188	1.29 (0.35–4.73); *p* = 0.702	0.80 (0.21–3.02); *p* = 0.743
Acute myocardial infarction	**5.04 (3.09–8.23); *p* < 0.001**	**3.50 (2.06–5.96); *p* < 0.001**	**2.58 (1.51–4.41); *p* < 0.001**	**1.98 (1.13–3.46); *p* = 0.017**
Pneumonia	**6.98 (4.88–9.97); *p* < 0.001**	**6.57 (4.52–9.56); *p* < 0.001**	**1.77 (1.26–2.48); *p* < 0.001**	**1.69 (1.20–2.39); *p* = 0.003**
Urinary tract infection	0.95 (0.66–1.36); *p* = 0.782	0.75 (0.50–1.12); *p* = 0.155	**1.40 (1.02–1.93); *p* = 0.038**	1.32 (0.93–1.86); *p* = 0.122
Fragility fracture	0.45 (0.16–1.27); *p* = 0.131	0.39 (0.14–1.14); *p* = 0.086	1.07 (0.54–2.12); *p* = 0.837	1.02 (0.51–2.07); *p* = 0.946

Reference group: no mortality after 1 year; ^+^ reference group: low frail risk; ^ reference group: Charlsons comorbidity index tertile 1; OR, odds ratio; CI, confidence interval; adjusted for age, gender, race, frailty status and age-adjusted Charlson comorbidity index; bold indicates significance (*p* < 0.05).

For late deaths compared to survivors, univariate analysis showed that age ≥ 85 years, intermediate frail risk, CCI score of more than 6, delirium, acute myocardial infarction (AMI), pneumonia and urinary tract infection (UTI) to be significantly associated with increased risk of mortality. In the multivariate analysis, risk factors for late death included age ≥ 85 years (OR 2.04, 95% CI 1.48–2.80), being at intermediate frail risk (OR 1.2, 95% CI 1.08–2.14), CCI score more than 6 (OR 1.98, 95% CI 1.37–2.87), delirium (OR 1.55, 95% CI 1.08–2.23), AMI (OR 1.98, 95% CI 1.13–3.46) and pneumonia (OR 1.69, 95% CI 1.20–2.39) (Table 2). Interestingly, while a diagnosis of delirium was not significantly associated with early death, it was associated with a higher risk of late death (OR 1.55, 95% CI 1.08–2.23).

Variables that retained statistical significance, and had an impact on the prediction model in the multivariate regression analyses for both early and long death were factored into our prediction model, namely age ≥ 85 years, pneumonia and AMI. Assigning 1 point for each positive variable, this risk score model had an AUC of 0.752 (95% CI 0.714–0.790, *p* < 0.001) for prediction of early death, and an AUC of 0.691 (95% CI 0.658–0.723, *p* < 0.001) for prediction of late death, respectively (Table 3). With inclusion of each additional positive predictor, the positive predictive value increased and sensitivity decreased. Higher scores were more predictive of mortality: patients with a score of 3 had a 54.3% chance of dying within 30 days, and a 71.4% chance of dying within 1 year.

**Table 3:** Prediction model for mortality from time of admission.

	Mortality within 30 days (early death)			Mortality within 1 year (late death)		
Score	1	2	3	1	2	3
Sensitivity	92.3	48.8	11.3	81.9	35.7	6.8
Specificity	43.8	84.8	98.5	47.8	87.0	98.8
PPV	20.7	33.7	54.3	40.5	54.3	71.4
NPV	97.3	91.2	87.5	85.9	75.7	71.0
Variables in model: age ≥ 85, pneumonia, acute myocardial infarction. PPV: positive predictive value; NPV: negative predictive value.						

Variables	Area under the curve	Standard error	Significance	95% confidence interval
(A) Comparison of individual risk predictors with the final model for early death				
Age ≥ 85	0.616	0.023	0.000	0.571–0.661
Pneumonia	0.710	0.023	0.000	0.666–0.754
AMI	0.571	0.026	0.003	0.521–0.621
All 3	0.752	0.019	0.000	0.714–0.790
(B) Comparison of individual risk predictors with the final model for late death				
Age ≥ 85	0.605	0.018	0.000	0.571–0.640
Pneumonia	0.631	0.018	0.000	0.595–0.666
AMI	0.553	0.018	0.003	0.517–0.589
All 3	0.691	0.016	0.000	0.658–0.723

## Discussion

Our study showed that nearly one in three older patients ≥75 years old admitted under internal medicine will die within one year which is similar to prior studies which showed that 24 to 28% will die within 1 year (13, 27). Previous mortality prediction tools focus on a single timepoint post-discharge (13, 14), whereas in our study we aimed to devise a tool that can predict early and later mortality, taking reference from an index admission. We found that patients who died within the year of discharge tended to be older, had a diagnosis of pneumonia, delirium, AMI, higher HFRS and CCI. Association with delirium was only evident in late mortality. Inclusion or exclusion of HFRS and CCI did not affect the overall predictive value of the model. Only three factors: ≥85 years old, diagnosis of pneumonia and AMI were included in our model with AUC ranging 0.691 to 0.752. By extracting the three variables of our prediction model and automating the risk computation for mortality, the electronic health system can potentially be able to alert physicians to patients’ estimated prognosis.

Our study findings are supported by existing literature. Amongst the top causes of death in older adults are cardiovascular diseases and pneumonia (28), which correspond to our findings. In particular, pneumonia makes up one-quarter of total deaths in older adults (28). Mortality rates for AMI are known to be higher in older age groups, even after adjusting for patient characteristics (29). Delirium is one of the most common complications in older general medicine patients (30, 31), but is often missed in clinical practice. Delirium is associated with more than three-fold mortality compared to non-delirious patients (32). Delirium as a risk factor for mortality was only significant in late deaths but not early deaths. This could be due to under reporting as we have previously showed that patients under geriatrician care were more frequently diagnosed with delirium (26.6%) than under other specialties (5.5%) (5). However, other studies have also shown that the effect of delirium on mortality appears to be delayed, with association seen with 12-month mortality (33) but not 30 days (34), as reflected in our data.

Both HFRS and CCI which are cumulative measures of comorbidities and known to influence mortality (20, 35) did not change the overall prediction for our study population. HFRS, while shown to be associated with poor outcomes (20), is not a proxy for function. Physical function or severe frailty defined by Clinical Frailty Scale or Fried’s phenotypic index have shown to be significantly associated with mortality (36, 37). One other possibility that both CCI and HFRS did not have an impact on the prediction model include patients in the extremes of age where complications related to comorbidities such as AMI may have a greater impact.

Our study discovered that opioid use was highest in cases of late deaths, surpassing that of early deaths. This observation suggests a potential lack of awareness of impending mortality and a strong emphasis on cure. In some centres, this could also be attributed to a lack of training or a relative scarcity of resources for palliative care (6, 38). Such circumstances may lead to a deficiency in necessary palliative measures for many individuals in their last month of life. This also impacts healthcare utilization. We found that those who died within 30 days had the longest length of stay (LOS) during their final admission and incurred the highest costs, followed by those who died within the year. These findings have been previously documented. LOS and cost are crucial metrics for healthcare providers and patients and are influenced by numerous factors. Importantly, physicians’ early subjective risk assessments play a significant role. In situations where physicians with varied clinical backgrounds and experiences are managing older patients, a standardized automated mortality prediction tool could be beneficial. It could prompt physicians to consider personalized care goals tailored to their patients’ prognosis, initiate early discussions on end-of-life care, and determine the right setting for care.

### Strengths and limitations

To the best of our knowledge, this is the first mortality prediction tool that is predictive over a range of timepoints post-discharge and can feasibly be fully automated and integrated into an electronic health system. Unlike previous prediction models for older adults, which required manual input and were time-consuming, our mortality prediction tool can derive all variables from patients’ electronic medical records. This allows for the bypassing of inconsistencies in the care of older adults that depend on physicians’ clinical experience, their subjective assessment, and their memory to consider life expectancy. As a result, workflows for appropriate care approaches such as advance care planning, home care, and reducing inappropriate prescribing can be implemented more uniformly.

However, our prediction model does have limitations. The accuracy of using ICD-10 codes depends on the expertise of junior doctors in documenting the discharge summary and the coder’s ability to translate clinical information from the case notes. The use of ICD-10 codes also fails to capture the severity of medical conditions and requires a longer lead time, as the individual would need to have the diagnostic coding done during a prior hospitalisation (39). The information of other factors which have impact on mortality such as functional ability, polypharmacy, social support or access to healthcare services were not available in our hospital database (40). Despite not having a parameter for function, our model still retained moderate prognostic performance as compared with other scales for older patients (41). We acknowledge that our three variables, while important and helps with usability of the prediction tool, may not capture the range of complexity of factors influencing mortality in older adults. As a result, our model may have limited predictive power, particularly in complex clinical scenarios where multiple factors interact to influence outcomes. In these instances, addition of further variables may be needed to enhance the accuracy and robustness of the prediction model. Furthermore, a small number of variables in the model may increase the risk of overfitting. Hence, our model needs to be tested on other cohorts, with possibility of including a larger number of predictors to improve generalizability.

## Conclusion

Older patients admitted to internal medicine service and died within one year from admission tend to be older, have diagnoses of pneumonia, delirium or AMI, and have higher HFRS and CCI scores. Our mortality prediction model using three easily derived variables – age, diagnosis of pneumonia and AMI – is a proof-of-concept that demonstrates the potential for electronic health systems to create automated medical alerts to guide physicians towards personalised care for older patients appropriate to their prognosis. More work is needed to validate this prediction model.

## Data availability statement

The data analyzed in this study is subject to the following licenses/restrictions: unfortunately we used a hospital based dataset which cannot be publicly released. Requests to access these datasets should be directed to NL, natalie_ling@nuhs.edu.sg.

## Ethics statement

The studies involving humans were approved by National Healthcare Group Domain Specific Review Board. The studies were conducted in accordance with the local legislation and institutional requirements. The ethics committee/institutional review board waived the requirement of written informed consent for participation from the participants or the participants’ legal guardians/next of kin because only de-identified data was used.

## Author contributions

VH: Conceptualization, Writing – original draft. NL: Conceptualization, Writing – review & editing. DA: Formal analysis, Writing – review & editing. YC: Formal analysis, Methodology, Writing – review & editing. RM: Conceptualization, Formal analysis, Supervision, Writing – review & editing.
