# Supplementary material for: Proof-of-concept for an automatable mortality prediction scoring in hospitalised older adults
Source: Front Med (Lausanne). 2024 May 23;11:1329107. doi: 10.3389/fmed.2024.1329107 (PMC11153690; doi:10.3389/fmed.2024.1329107)
Supplement: Supplementary file 1 [file Table_1.docx]

**Supplementary Table 1.** Demographics in inpatient mortality and mortality within 30 days

|  | Inpatient | Within 30 days | **P-Value** |
| --- | --- | --- | --- |
|  | n = 105 (62.5%) | n = 63 (37.5%) |  |
| **Demographics** |  |  |  |
| Gender |  |  | 0.523 |
| Male | 52 (49.5) | 28 (44.4) |  |
| Female | 53 (50.5) | 35 (55.6) |  |
| Age (years) | 87.63 ± 6.76 | 87.54 ± 6.18 | 0.932 |
| Ethnicity |  |  | 0.360 |
| Chinese | 88 (83.8) | 54 (85.7) |  |
| Malay | 7 (6.7) | 7 (11.1) |  |
| Indian | 6 (5.7) | 1 (1.6) |  |
| Others | 4 (3.8) | 1 (1.6) |  |
| Diagnosis |  |  |  |
| Pneumonia | 81 (77.1) | 29 (46.0) | **<0.001** |
| Delirium | 37 (35.2) | 29 (46.0) | 0.166 |
| Fragility Fracture | 1 (1.0) | 3 (4.8) | 0.117 |
| Urinary Tract Infection | 27 (25.7) | 23 (36.5) | 0.139 |
| Stroke | 1 (1.0) | 0 (0.0) | 0.437 |
| Intracranial Bleed | 2 (1.9) | 0 (0.0) | 0.270 |
| Acute Myocardial Infarction | 25 (23.8) | 9 (14.3) | 0.137 |
| Comorbidities |  |  |  |
| Diabetes | 40 (38.1) | 30 (47.6) | 0.225 |
| Hypertension | 47 (44.8) | 27 (42.9) | 0.810 |
| Hyperlipidaemia | 29 (27.6) | 26 (41.3) | 0.068 |
| Dementia | 16 (15.2) | 10 (15.9) | 0.912 |
| Chronic Kidney Disease | 48 (45.7) | 21 (33.3) | 0.114 |
| Serum Albumin | 28.40 ± 5.38 | 30.08 ± 5.12 | 0.093 |
| Hospital Frailty Risk Score (median(IQR)) | 6.40 (11.90) | 8.50 (10.70) | 0.282 |
| Low | 42 (40.0) | 19 (30.2) | 0.424 |
| Intermediate | 43 (41.0) | 29 (46.0) |  |
| High | 20 (19.0) | 15 (23.8) |  |
| Age adjusted Charlson’s Comorbidity Index (median(IQR)) | 6.00 (3.00) | 6.00 (3.00) | 0.315 |
| Tertile 1 | 48 (45.7) | 27 (42.9) | 0.072 |
| Tertile 2 | 26 (24.8) | 25 (39.7) |  |
| Tertile 3 | 31 (29.5) | 11 (17.5) |  |
| Fentanyl | 13 (12.4) | 8 (12.7) | 0.952 |
| Morphine | 3 (2.9) | 6 (9.5) | 0.063 |
| **Outcomes** |  |  |  |
| Number of admissions in past 1 year |  |  |  |
| Mean | 1.44 ± 1.69 | 1.63 ± 1.89 | 0.485 |
| Median (IQR) | 1.00 (2.00) | 1.00 (3.00) | 0.568 |
| Length of stay in final admission (Days) |  |  |  |
| Mean | 8.51 ± 8.25 | 13.00 ± 9.92 | **0.003** |
| Median (IQR) | 7.00 (9.00) | 9.00 (12.00) | 0.087 |
| Total Cost ($) |  |  |  |
| Mean | 6882.69 ± 6273.92 | 9838.17 ± 7624.65 | **0.007** |
| Median (IQR) | 4817.44 (7278.81) | 7751.99 (9043.49) | **<0.001** |

Values presented as n (%) or mean ± SD or median (IQR) whenever specified; Bold indicates significant difference (p <0.05); ^abc^ Values with common superscript alphabet are significantly different.
